# Long-term exercise adaptation. Physical aging phenomena in biological networks

**DOI:** 10.3389/fnetp.2023.1243736

**Published:** 2023-10-04

**Authors:** Robert Hristovski, Natàlia Balagué, Marko Stevanovski

**Affiliations:** ^1^ Faculty of Physical Education, Sport and Health, University “Ss. Cyril and Methodius”, Skopje, North Macedonia; ^2^ Complex Systems in Sport Research Group, Institut Nacional d’Educació Fisica de Catalunya (INEFC), Universitat de Barcelona (UB), Barcelona, Spain

**Keywords:** heterochronicity, memory, rejuvenation, physical aging, entropy barrier, energy barrier, complex networks, strong anticipation

## 1 Introduction

Long-term adaptation to exercise, consisting of the cumulative, delayed, and residual effects (Issurin, 2009), is a consequence of task-dependent functionally integrated physiological networks and their interaction with the environment. However, principles of dynamic integration of these networks remain partially unknown (Ivanov and Bartsch, 2014; Balagué et al., 2020; Ivanov et al., 2021a; Ivanov et al., 2021b; Ivanov, 2021; Rogers, Mourot, Doucende, and Gronwald, 2021; Romero-Ortuño et al., 2021; Garcia-Retortillo and Ivanov, 2022). Particularly, the long-term effects of exercise on network dynamics are a much less researched area (e.g., Balague et al., 2016; Vázquez et al., 2016). A long-recognized general characteristic to the dynamics of the long-term effects is the *tendency* toward the slowing down of fitness^1^ record value^2^ occurrence, on average, on a large variety of time scales (e.g., Platonov, 1988). Moreover, the long-term rate of decay of the achieved fitness level, after temporary or permanent cessation of exercise, is *inversely* proportional to the accumulated training time (e.g., Zatsiorsky and Kraemer, 2006). This highly reproducible effect has been known since Hettinger’s early works on isometric strength (Hettinger, 1966). “Soon ripe, soon rotten” is a common catchphrase that depicts this effect. The causes of this phenomenon, however, are still unknown (Gavanda, Geisler, et al., 2020). In this opinion paper, our aim is to suggest that the previously mentioned phenomena may be successfully, theoretically, and methodologically approached by conceptualizing them as multilevel-physiological-network *aging phenomena*, known from condensed matter physics (see, e.g.,; Bouchaud, 2000)^3^. Before explicitly discussing this possibility, we briefly build an argument for our case.

Supercompensation, the process of increasing the substrate and functional possibilities of biological systems above the levels that precede an acute exercise perturbation, is the most fundamental multilevel process that underpins exercise-induced long-term adaptation. Recently, it has been conceptualized in terms of a *strong anticipation phenomenon* (Hristovski and Balagué, 2020). With this respect, important developments suggest that this kind of adaptive anticipatory process can be understood in terms of multiplicative cascades synchronized to the statistical structure of the environmental dynamics (Stephen and Dixon, 2011). Multiplicative cascades were recently discussed as formal characterization of anomalous diffusion processes (Mangalam, Metzler, and Kelty-Stephen, 2023), which are ubiquitous in hierarchical energy/entropy landscapes, characteristic for aging complex systems with many metastable states (Bouchaud, 2000). Complex, and by extension biological, networks may contain a very large number of metastable states. Metastable states are states in which the network may reside for a prolonged time but which eventually are being abandoned. Metastability has already been shown to exist in many types of biological networks, e.g., neural, transcriptomic, and endocrine networks (see Gili, Ciullo, Spalletta, 2018; Helmling, C., Klötzner, et al., 2018; Avila-Varela, Hidalgo-Lopez, et al., 2023). The metastable states in biological systems are constrained by the interactions between a vast number of multiscale co-adaptive networks, such as neuromuscular, fascial, cardiorespiratory, hormonal, immune, and the -omics networks, in cells, blood, etc. (Balagué et al., 2020). One of the main consequences of the heterogeneity of multiscale networks is the wide difference of time-scales of different variables that influence the biological network dynamics and, hence, the nested landscape of network-wide metastable states (i.e., minima), in which the fitness level may be temporarily trapped (Bouchaud, 2000). The metastable states are separated by energy/entropy barriers^4^. The depth of the trapping minima is proportional to the height of the barriers that surround them. On the other hand, the height of the barriers is roughly proportional to the quantity of stabilized reconfigurations that occurred in the network during its evolution. In this view, exercise-induced fitness can be considered a strong anticipation-based collective variable (Hristovski et al., 2010) generated by heterogenous network-wide hierarchical multiplicative cascade processes each time it is measured.

## 2 A heuristic model of the exercise-induced aging process

*Aging* is manifested in two basic facts: 1) The longer the fitness evolves (i.e., ages) under a certain sequence of perturbations (i.e., exercise stimuli), the more it slows down; and 2) the longer the fitness variables evolve, the longer the achieved fitness effects last after the cessation of the perturbations. Aged variables become ‘inert’ and resist change either for further enhancement or decay. The aging of the fitness variables can be measured by some macroscopic variables, that is, global fitness measure (e.g., max-power, strength, endurance, skill-related variables etc.), and/or some network-specific (e.g., network excitability, connectivity, efficiency, or energy flux) fitness variables. Aging is reflected in the increasingly longer waiting times for the record values of the fitness variable (Jensen and Sibani, 2013), or more technically as $\dot{\mu }\propto at^{-1}$ , where $\dot{\mu }$ represents the rate of decay of the average number of record values of the variable per unit time *t* and a represents a constant.

Hence, in our heuristic model, aging is an exercise-activated process of relaxation (equilibration) of metastable multilevel-network configurations (see Bouchaud, 2000 for details). The core of this model includes the *mutual parametrizing interactions* of processes that relax on different characteristic time-scales. This means that, after some exercise time, the network’s faster evolving variables (e.g., enzymatic or signaling network activity, neural excitability, etc.) may have already relaxed and stabilized, the more slowly evolving variables (e.g., muscle; tendon; bone and heart remodeling; intra-, inter muscle, and interlimb coordination; capillarization, etc.) have not yet. Then, the stabilized network processes *parametrize* (i.e., constrain) the dynamics of the slower processes. As a result, hierarchical, network-wide, adaptive processes are being trapped, i.e., stabilized, between increasingly higher energy/entropy barriers (Bouchaud, 2000). Consequently, the response to exercise perturbations often decelerates and plateaus (Gorostiaga, Izquierdo, et al., 1999).

Plateauing (Gorostiaga et al., 1999) is captured phenomenologically by general, experimentally found, decelerating (e.g., logarithmic) laws of learning (Harris, 2022). Hence, the decelerating scenario of fitness growth as relaxation over hierarchical energy and/or entropy landscape (Bouchaud, 2000) directly predicts empirically detected laws. In order to enhance the system further, one must exert a change in the perturbations, e.g., directed variation in the load. The ultimate plateauing phases of the aging process may be detected on different time scales, from novices who practice their first weeks of exercise to individuals who exercise for decades (Platonov, 1988).

### 2.1 Rejuvenation and memory effects

Increasing the specificity (directed reduction of the variability^5^ of exercise perturbations) (Gamble, 2006) can initiate a fitness increase (on scales of months to decades). This is exactly what the phenomenon of *rejuvenation* means in physically aging systems. By reducing the variability of perturbations, i.e., increasing their specificity, the previously acquired coarser organization network modes stay stable, but more specific detailed configurational refinements are being activated (see Bouchaud, 2000). The finer structure of the network energy/entropy landscape is being revealed. The system restarts the aging process. Here, the measure of susceptibility (receptivity) χ may be used, which is defined as a change in some fitness function $\left(f\right)$ per constant unit change in the accumulated exercise perturbations $\left(p\right)$ , or $\chi =\frac{df\left(p\right)}{dp}$ . As a consequence of the increase in the specificity, the susceptibility to the new specific perturbations again increases, which further improves the values of the fitness function $\left(f\right)$ . This is usually an effect of exploring the more detailed structure of the landscape of task-specific physiological/biomechanical metastable configurations (Stone et al., 2022). When variability of perturbations is returned to the previous (less specific and more variable) level, the susceptibility to exercise perturbations χ returns to the values characteristic for the states before the increment of the exercise specificity. The network energy/entropy landscape recovers the pre-rejuvenation coarser form. As a consequence, when measured, the system is still able to organize the previously stabilized coarser, less specific, structure (as in the bicycling skill). This is called a *memory* effect in physically aging systems (Bouchaud, 2000). Likely, different modalities of transfer may show different influences on network rejuvenation and memory effects (Brearley and Bishop, 2019). It is also possible that rejuvenation and memory effects exist only in some fitness variables, and detecting these variables would provide information about the structure of the biological networks.

### 2.2 The detraining effects of aging

Detraining effects of aging are measured starting from the highest fitness value achieved after switching-off of perturbations that acted on the system while it aged for time *t*_*e*_. The main effect of aging detraining dynamics can be written down in both a stretched exponential form $f\left(t_{e,}t_{e}+t\right)\propto e^{{-\left(\gamma \left(t_{e}\right)t\right)}^{p\left(t_{e}\right)}}$ and power law form $f\left(t_{e,}t_{e}+t\right)\propto at^{\left(-pt_{e}\right)}$ , where *f(t*_*e,*_ *t*_*e*_ *+t)* represents the fitness function, *t* represents detraining time, a is a constant which may depend on *t*_*e*_, and constants γ and *p* depend on *t*_*e*_ and control the rate of decay of the fitness function.

The longer the aging process, i.e., the accumulated exercise time (*t*_*e*_) of the system, the longer it takes the system to lose its memory of the past states, acquired during aging and *vice versa*. This very effect is typical in long-term detraining effects (e.g., Hettinger, 1966; Zatsiorsky and Kraemer, 2006). In the theoretical framework of physical aging, this results from the network being trapped in a deep minimum (i.e., the exceedingly stable state) due to the accumulated aging of the slow variables (Jensen and Sibani, 2013). As a consequence, escaping from that state would require much more time, proportional to the quantity of the required reverse (detraining) reconfigurations of the network, compared to the time required to escape from much shallower minima (i.e., less stable states), characteristic for networks with less accumulated exercise time (*t*_*e*_). However, the biological reasons for this stabilization are yet unknown (Gavanda, Geisler, et al., 2020).

For some fitness components (e.g., strength), one may attribute this effect, at least partially, to the phenomenon of muscle memory (see Sharples, and Turner, 2023). Skeletal muscle memory, which is hypothesized to comprise the synergistic action of cellular (myonuclear) and epigenetic network processes responsible for hypertrophy, would enable the quick recovery of fitness function (e.g., strength) during retraining, after longer periods of detraining. However, in Hettinger (1966), the smaller the maximal acquired strength and the longer it has been acquired (larger accumulated exercise time), the slower was the rate of its decay during the detraining period and *vice versa*. In other words, it was not the maximal level of acquired strength and hypertrophy but the accumulated exercise time, which had a *positive* effect on the strength stability during the detraining period. Certainly, longer accumulated exercise time often brings about a higher level of strength as well. However, the said work shows that, when partialized with respect to the acquired maximal strength level, the accumulate exercise time, alone, is the generator of the strength stability. Thus, the following question is raised: How does the accumulated exercise time (*t*_*e*_) stabilize the multilevel-network processes, including possible myonuclear and epigenetic mechanisms, which positively contribute to the fitness stability during detraining?

Moreover, the “soon ripe soon rotten” phenomenon is also present in the domain of skill acquisition (e.g., Yin et al., 2009), where the early acquired skill is easily subject to decay, but after prolonged use, it may last a lifetime. Hence the said phenomenon cannot be explained by the same cellular processes/mechanisms as muscular hypertrophy. In this respect, different aging phenomena may consist of different local processes/mechanisms but may also have common mechanisms. Hence, we hypothesize that it is the mutually parametrizing (constraining) role of multi-network fast and slow processes, which is the *general principle* that generates the diverse physical aging phenomena.

## 3 Research program

The heuristic model described previously remains largely qualitative, and to become quantitatively predictive, a research program focused (though not exclusively) on the following directions is required: 1. Establishing the *essential network variables* and their characteristic time-scales at each organism level (e.g., rate constants, connectivity measures, reciprocally compensating, i.e., synergic couplings, etc.), which potentially age and rejuvenate. In our opinion, the best approach would be the research on the *long-term evolution* of a macroscopic fitness variable (e.g., strength) and one or more nested networks associated with the macroscopic variable (see, e.g., for muscle-network connectivity measures during acute fatigue Garcia-Retortillo et al., 2023); 2. determination of networks’ energy/entropy landscapes; for example, the landscape of connectivity measures of intermuscular or endocrine interactions; 3. research on the *dynamic mechanisms* of aging of synergies between multilevel-network processes; 4. empirical and modeling work on the *heterochronicity* of multilevel network variables that age and rejuvenate and, hence, constrain the reconfiguration process of the physiological network fitness level at different time-scales. It is highly likely that heterochronicity has large interindividual variability and different organic systems, so the networks may have a strong individual imprint; 5. how the change in the variability of exercises (less-more specificity) affects the multilevel aging and rejuvenation of network configurations; 6. defining the multilevel network bio-markers that can explain and assess the long-term exercise-induced fitness effects of aging, rejuvenation, and memory. This research program requires effort and time, but in the long run, it may prove beneficial for science and the society.

## Footnotes

1 Under “fitness,” here, we understand any exercise-induced adaptation in components such as strength, power, types of endurance, speed, complex perceptual-motor skills, and flexibility, as well as the generalized adaptations which emerge from the interactions among these components.

2 The record value is the highest value measured in some fitness variables in some period of time for a certain individual.

3 The concept of “aging,” in this paper, refers to the phenomenon of “physical aging” present in, e.g., complex disordered systems. It does *not* refer to human biological aging. The reader should not conflate both phenomena.

4 In networks, the term energy barrier may or may not refer to physical energy. It may refer simply to the difficulty of overcoming some state. Entropy barriers, on the other hand, correspond to the possibilities (paths) that the system can use to escape the current metastable state. The more paths are afforded, the smaller is the entropy barrier and the higher is the probability of escaping and switching to another metastable state (see, e.g., Jensen and Sibani, 2013).

5 In the general formulation of the Fokker–Planck equation, the *variance* corresponds to the temperature term in physical systems.
